# Critical appraisal of AMSTAR: challenges, limitations, and potential solutions from the perspective of an assessor

**DOI:** 10.1186/s12874-015-0062-6

**Published:** 2015-08-13

**Authors:** Clovis Mariano Faggion

**Affiliations:** Department of Periodontology, Faculty of Dentistry, University of Münster, Waldeyerstraße 30, 48149 Münster, Germany

**Keywords:** AMSTAR checklist, Systematic review, Methods, Clinical evidence

## Abstract

**Background:**

Systematic reviews are pivotal components in the development of evidence-based clinical guidelines. To evaluate the methodological quality of these systematic reviews, several tools have been proposed. Among them, the assessment of multiple systematic reviews (AMSTAR) checklist is probably used most frequently. This tool comprises 11 items related to the steps taken when conducting a systematic review, and it is claimed to have good face and content validity. The objective of this debate paper was twofold: (a) to critically evaluate the ability of all AMSTAR checklist items to adequately determine the methodological quality of a systematic review; and (b) to describe difficulties regarding interpretation of the checklist, and provide potential solutions for these difficulties.

**Discussion:**

Some items of the AMSTAR checklist seem to assess the quality of reporting of a systematic review more than its methodological quality. For example, item 7 may not “capture” the true methodological quality of primary studies included in the systematic review. Item 10 does not likely result in the collection of in-depth information on the presence of publication bias in the systematic review. Furthermore, some items may be difficult to interpret, hindering accurate assessment. For example, item 5 does not explicitly indicate whether a list of documents excluded in each phase of selection (i.e., after evaluation of titles and abstracts, and after full-text assessment) should be reported.

**Summary:**

The present debate paper evaluated and discussed some methodological limitations of the AMSTAR checklist, as well as challenges involved in evaluation of the checklist’s items. Several suggestions are also made to optimize the use of this checklist. The information in this paper may stimulate further discussion among systematic reviewers, methodologists and clinicians.

## Background

Systematic reviews (SRs) are important components in the decision-making process in clinical practice. For example, SRs of randomized controlled trials (RCTs) form the basis for the development of clinical guidelines for interventions. Thus, evaluation of the methodological quality of a systematic review is crucial to avoid the development of a clinical guideline based on potentially misleading information.

Several tools have been developed to evaluate the methodological quality of a systematic review. Currently, the assessment of multiple systematic reviews (AMSTAR) checklist [1] appears to be among the most frequently used tools. A quick search of MEDLINE (via PubMed) using the key word “AMSTAR” on 14 March 2015 retrieved 200 documents, most of which described the use of this checklist to evaluate the methodological quality of SRs published in a great variety of medical disciplines. The AMSTAR checklist comprises 11 items related directly to the necessary steps taken when performing a systematic review. It is claimed to have good face and content validity for measuring the methodological quality of SRs that include only RCTs [1]. Currently, AMSTAR authors are developing a version of the checklist for the evaluation of SRs including non-randomized trials [2].

Although the AMSTAR checklist has been validated and used consistently since its publication in 2007, differentiation of whether it evaluates the quality of reporting or the methodological quality of a systematic review can be difficult. Quality of reporting is related strictly to what is reported in the published text, and thus increases with the completeness of an article. Methodological quality is a broader term that involves different concepts such as internal, external, construct, and descriptive validity [3]. Nevertheless, the ways in which AMSTAR items were developed and have been evaluated raise questions about whether this tool can “capture” the methodological quality of SRs evaluated. In addition, although the authors of the AMSTAR checklist explicitly state the rationale for the inclusion of each item, clear guidance on the use of the items to evaluate a systematic review is lacking in some cases.

AMSTAR checklist items are presented in the form of questions, with possible responses of YES (item/question fully addressed), NO (item/question not addressed), CANNOT ANSWER (not enough information to answer the question), and NOT APPLICABLE. The AMSTAR checklist was not originally intended to provide quantitative scores, and one can argue that this more subjective evaluation of items might reduce the reliability and replicability of measurements. Another group of researchers developed a revision of the AMSTAR checklist (R-AMSTAR) with quantitative item scoring [4]. However, adequate weighting of items according to relative importance to provide a final score is arguably difficult. Some evidence suggests that the measurement properties of the R-AMSTAR need to be studied further [5]. Thus, the question of whether the content validity of the checklist should involve quantitative measures remains open. The present work focuses exclusively on the AMSTAR checklist.

The main purpose of this debate paper is to critically evaluate the ability of all AMSTAR checklist items to adequately determine the methodological quality of a systematic review. A secondary objective is to describe challenges regarding interpretation of the checklist, and present potential solutions to these challenges. AMSTAR checklist items and guiding notes (taken from the AMSTAR home page) [2] are presented below in italics, followed by critical evaluations. The notes do not appear on the original checklist [1].

## Discussion

*1. Was an ‘a priori’ design provided? The research question and inclusion criteria should be established before the conduct of the review. Note: Need to refer to a protocol, ethics approval, or pre-determined/a priori published research objectives to score a “yes.”*

The text of the item appears to be clear. When a systematic review does not describe the research question and inclusion criteria, the assessor should record a NO response. However, the note is not sufficiently clear for adequate evaluation, as reference to “pre-determined/a priori published research objectives” is sufficient for a score of YES. What about the reporting of detailed eligibility criteria? This item is important for the evaluation of potential deviations in the protocol, which would imply in some sort of “selective outcome reporting” [6]. Thus, to clarify this item, the note should also require reference to a priori inclusion/exclusion criteria.

*2. Was there duplicate study selection and data extraction? There should be at least two independent data extractors and a consensus procedure for disagreements should be in place. Note: 2 people do study selection, 2 people do data extraction, consensus process or one person checks the other’s work.*

Consensus means that assessors reach general agreement on a specific issue, whereas the note states that this requirement can be fulfilled when one assessor “checks the other’s work.” Thus, this explanation implies that consensus must not necessarily be achieved, in apparent contradiction of the item 2 text. One may argue that only checking the work of the first assessor may bias the evaluation of the assessor performing this check. Therefore, the phrase “or one person checks the other’s work” should be deleted from the note to resolve this issue.

*3. Was a comprehensive literature search performed? At least two electronic sources should be searched. The report must include years and databases used (e.g., Central, EMBASE, and MEDLINE). Key words and/or MESH terms must be stated and where feasible the search strategy should be provided. All searches should be supplemented by consulting current contents, reviews, textbooks, specialized registers, or experts in the particular field of study, and by reviewing the references in the studies found. Note: If at least 2 sources + one supplementary strategy used, select “yes” (Cochrane register/Central counts as 2 sources; a grey literature search counts as supplementary).*

Because lists of publications do not match perfectly among databases [7], a search of at least 2 electronic databases seems to be important to increase comprehensiveness (interestingly, new evidence suggests that the benefits for searching sources beyond one major database such as MEDLINE may be modest [8]). Supplementary strategies may complement the potential lack of comprehensiveness of electronic searches. This item does not clearly identify “hand-searching” as a supplementary strategy. Hand-searching, as defined by the Cochrane Collaboration, is the “planned searching of a journal page by page (i.e. by hand), including editorials and letters, to identify all reported RCTs and NON-RCTs” [7]. Thus, hand-searching should be listed explicitly in the item. Furthermore, the number of supplementary strategies seems to be selected arbitrarily. Data on the minimum number of such sources required for a comprehensive search strategy are probably lacking, but comprehensiveness would arguably increase with the number of supplementary sources searched. Thus, to increase comprehensiveness, use of at least 2 supplementary strategies should be recommended.

*4. Was the status of publication (i.e. grey literature) used as an inclusion criterion? The authors should state that they searched for reports regardless of their publication type. The authors should state whether or not they excluded any reports (from the systematic review), based on their publication status, language etc. Note: If review indicates that there was a search for “grey literature” or “unpublished literature,” indicate “yes.” SINGLE database, dissertations, conference proceedings, and trial registries are all considered grey for this purpose. If searching a source that contains both grey and non-grey, must specify that they were searching for grey/unpublished lit.*

This item seems to be informative and clear. The main concern is the number of sources used to search the grey literature. The question of whether a search of a single source results in a sufficiently comprehensive retrieval of unpublished information remains. Thus, as stipulated in item 3 for the searching of electronic databases, this item should recommend that at least 2 sources be searched to meet this criterion, to increase the comprehensiveness of searches for unpublished material.

*5. Was a list of studies (included and excluded) provided? A list of included and excluded studies should be provided. Note: Acceptable if the excluded studies are referenced. If there is an electronic link to the list but the link is dead, select “no.”*

In a systematic review, the process of document selection (inclusion and exclusion) is performed in two phases: (a) the evaluation of titles and abstracts, followed by (b) the evaluation of full texts. In these 2 phases, documents that meet the eligibility criteria are included and those that do not are excluded. This checklist item does not clearly indicate whether the list of studies should be derived from the first or second phase. To improve transparency and clarity, the checklist should make this differentiation. Most SRs involving large numbers of initially selected papers report a full list of papers excluded after full-text evaluation. Nevertheless, to enable reproducibility of all systematic review steps, documents excluded in the first and second phases of selection should be listed, with reasons for exclusion. Most scientific journals now allow authors to provide such information, which is often lengthy and cannot be published in printed format, in online appendices.

*6. Were the characteristics of the included studies provided? In an aggregated form such as a table, data from the original studies should be provided on the participants, interventions and outcomes. The ranges of characteristics in all the studies analyzed e.g., age, race, sex, relevant socioeconomic data, disease status, duration, severity, or other diseases should be reported. Note: Acceptable if not in table format as long as they are described as above.*

Among AMSTAR items, this item probably receives the largest number of YES responses across disciplines. Most SRs include tables describing the features of primary studies included. The challenge related to this item is the establishment of a threshold for the minimum amount of information (i.e., number of characteristics) required for a YES response. Such a threshold could be established, with characteristics that are applicable in most disciplines, to facilitate the use of the item and increase the homogeneity of assessment. Nevertheless, this strategy might imply less comprehensive evaluation due to the lack of opportunity for more subjective judgments, although less experienced systematic reviewers will benefit from more defined and clearer guidance.

*7. Was the scientific quality of the included studies assessed and documented? ‘A priori’ methods of assessment should be provided (e.g., for effectiveness studies if the author(s) chose to include only randomized, double-blind, placebo controlled studies, or allocation concealment as inclusion criteria); for other types of studies alternative items will be relevant. Note: Can include use of a quality scoring tool or checklist, e.g., Jadad scale, risk of bias, sensitivity analysis, etc., or a description of quality items, with some kind of result for EACH study (“low” or “high” is fine, as long as it is clear which studies scored “low” and which scored “high”; a summary score/range for all studies is not acceptable).*

The main concern with this item is whether good reporting represents true methodological quality. For example, one may argue that inclusion of a “description of quality items, with some kind of result” does not represent adequate evaluation of primary studies. Some tools for evaluation of the quality of evidence listed in this item, such as the Jadad scale [9], have received criticism because they seem to be related more to reporting than to quality. Furthermore, the Jadad scale contains no mention of allocation concealment, which is important in the evaluation of an RCT’s internal validity. Another situation of concern is authors’ use of the incorrect tool to evaluate primary studies, such as the application of a tool used to evaluate RCTs to the evaluation of studies with other designs, such as retrospective cohort studies. At times, systematic review authors include primary studies with several designs; in these cases, the appropriate tool should be used for each type of study. Thus, the tool (s) used to evaluate the quality/risk of bias (ROB) of primary studies included in a systematic review should be sufficiently comprehensive to reflect the true quality of these studies. One such instrument is the Cochrane Collaboration’s ROB tool [10], which enables the evaluation of important domains related directly to the internal validity of an RCT and may capture the strengths and weaknesses of primary studies included. Another tool is the GRADE approach [11], which enables evaluation of the overall quality of evidence and includes ROB among the domains assessed. The GRADE approach is becoming universally used and has been proposed for the development of evidence-based guidelines [12]. Acceptance of these tools as standards for the evaluation of primary trials would increase the homogeneity of comparison/evaluation among SRs from the same discipline.

*8. Was the scientific quality of the included studies used appropriately in formulating conclusions? The results of the methodological rigor and scientific quality should be considered in the analysis and the conclusions of the review, and explicitly stated in formulating recommendations. Note: Might say something such as “the results should be interpreted with caution due to poor quality of included studies.” Cannot score “yes” for this question if scored “no” for question 7.*

This item demonstrates the importance of using a good approach for item 7, as the 2 items are interconnected. When authors fail to clearly report the quality measures mentioned in item 7, item 8 will be assigned a NO response. Although detailed information about the quality of primary studies may be difficult to convey in a systematic review, the use of sentences such as “the results should be interpreted with caution due to poor quality of included studies” may not be sufficient. Thus, the checklist could be improved by establishing a minimum (and higher-level) threshold for the reporting of detailed information, such as by requiring more specific information from systematic review authors on (a) how the quality/ROB of studies affected treatment effect estimates; and (b) whether the quality/ROB scores were incorporated in meta-analytic estimates (for meta-analyses). If authors did not incorporate the quality/ROB in the results, they should provide an explanation.

*9. Were the methods used to combine the findings of studies appropriate? For the pooled results, a test should be done to ensure the studies were combinable, to assess their homogeneity (i.e., Chi-squared test for homogeneity, I2). If heterogeneity exists a random effects model should be used and/or the clinical appropriateness of combining should be taken into consideration (i.e., is it sensible to combine?). Note: Indicate “yes” if they mention or describe heterogeneity, i.e., if they explain that they cannot pool because of heterogeneity/variability between interventions.*

This item reflects a good rationale: when conducting SRs, assessors should first evaluate the feasibility of combining results in a meta-analysis. Then, they should consider the statistical test performed by the authors to determine the degree of heterogeneity. In the presence of heterogeneity, a random model should be used and heterogeneity should be evaluated at the clinical level. Then, assessors should determine whether systematic review authors conducted meta-analyses properly. The concern with this item is the recommendation to assess the use of a random model “and/or” evaluation of clinical heterogeneity, which allows for item fulfillment based on accounting for only one type of heterogeneity. Although statistical heterogeneity may also involve clinical heterogeneity [13], the assessor should carefully evaluate the presence of both types of heterogeneity in determining the sensibility of combining results. Thus, the word “or” should be deleted from the sentence.

*10. Was the likelihood of publication bias assessed? An assessment of publication bias should include a combination of graphical aids (e.g., funnel plot, other available tests) and/or statistical tests (e.g., Egger regression test, Hedges-Olken). Note: If no test values or funnel plot included, score “no”. Score “yes” if mentions that publication bias could not be assessed because there were fewer than 10 included studies.*

This item is another case in which the quality of reporting should not be used as a proxy for methodological quality. For example, the item indicates that the authors of a meta-analysis including fewer than 10 studies are not obligated to report any statistical examination of publication bias, very likely because of the “lack of power” of statistical tests to detect this bias [14]. Thus, reporting on non-evaluation of the risk of publication bias because of a small sample constitutes fulfillment of this item. However, the systematic review is not necessarily free of publication bias. Thus, “high-quality” reporting may not be translated directly into high methodological quality.

The first sentence of this item should be changed to “was the systematic review at high risk of publication bias?” This revision would lead to evaluation based on authors’ description of this risk (e.g., as high or low), even in SRs involving small numbers of studies. In other words, a YES response would require systematic review authors to report perceived risk of publication bias, with a strong argument/rationale to support this statement.

*11. Was the conflict of interest included? Potential sources of support should be clearly acknowledged in both the systematic review and the included studies. Note: To get a “yes,” must indicate source of funding or support for the systematic review AND for each of the included studies.*

This item does not clearly indicate whether the conflict of interest (COI) of a researcher or any person directly involved in a systematic review should be reported. The question “was the conflict of interest included?” does not specify who should declare the COI, and the note suggests that reporting of sources of funding or support for the systematic review and primary studies is sufficient. Thus, this item should be clarified to enable more in-depth assessment. A YES response should require reporting of COI for all persons involved in the studies and sources of funding. COI involves, for example, a strong relationship between study participants and the enterprise(s) supporting the study (e.g., researchers owning stocks in a company or serving on its board).

## Summary

This debate paper presents a critical appraisal of the characteristics of the AMSTAR checklist, a validated and recognized tool for the evaluation of the methodological quality of SRs. Some challenges of assessment have been described from the point of view of the assessor, and potential solutions to improve the reliability of the tool and homogeneity of its use have been offered. The paper will ideally generate further discussion among systematic reviewers and methodologists aiming to optimize the use of the AMSTAR checklist.

## Abbreviations

AMSTAR: the assessment of multiple systematic reviews
SRs: Systematic reviews
RCTs: Randomized controlled trials
R-AMSTAR: Revised AMSTAR
ROB: Risk of bias
GRADE: Grading of Recommendations Assessment, Development and Evaluation
CI: Conflict of interest

## References

[1] Shea BJ, Grimshaw JM, Wells GA, Boers M, Andersson N, Hamel C (2007). Development of AMSTAR: a measurement tool to assess the methodological quality of systematic reviews. BMC Med Res Methodol.

[2] AMSTAR. Assessing the Methodological Quality of Systematic Reviews. http://amstar.ca/. Acessed 26 March 2015.

[3] Farrington DP (2003). Methodological quality standards for evaluation research. Ann Am Acad Polit Soc Sci.

[4] Kung J, Chiappelli F, Cajulis OO, Avezova R, Kossan G, Chew L (2010). From systematic reviews to clinical recommendations for evidence-based health care: validation of Revised Assessment of Multiple Systematic Reviews (R-AMSTAR) for grading of clinical relevance. Open Dent J.

[5] Pieper D, Buechter RB, Li L, Prediger B, Eikermann M (2015). Systematic review found AMSTAR, but not R(evised)-AMSTAR, to have good measurement properties. J Clin Epidemiol.

[6] Higgins JPT, Altman DG, Sterne JAC (editors). Chapter 8: Assessing risk of bias in included studies. In: Higgins JPT, Green S (editors). Cochrane handbook for systematic reviews of interventions version 5.1.0 (updated March 2011). The Cochrane Collaboration, 2011. Available from www.cochrane-handbook.org.

[7] Lefebvre C, Manheimer E, Glanville J. Chapter 6: Searching for studies. In: Higgins JPT, Green S. Cochrane handbook for systematic reviews of interventions version 5.1.0 (updated March 2011). The Cochrane Collaboration, 2011. Available from www.cochrane-handbook.org.

[8] Halladay CW, Trikalinos TA, Schmid IT, Schmid CH, Dahabreh IJ. Using data sources beyond PubMed has a modest impact on the results of systematic reviews of therapeutic interventions. J Clin Epidemiol. 2015; http://dx.doi.org/10.1016/j.jclinepi.2014.12.017 10.1016/j.jclinepi.2014.12.017 26279401

[9] Jadad AR, Moore RA, Carroll D, Jenkinson C, Reynolds DJ, Gavaghan DJ (1996). Assessing the quality of reports of randomized clinical trials: is blinding necessary?. Control Clin Trials.

[10] Higgins JP, Altman DG, Gøtzsche PC, Jüni P, Moher D, Oxman AD (2011). The Cochrane Collaboration’s tool for assessing risk of bias in randomised trials. BMJ.

[11] Guyatt G, Oxman AD, Akl EA, Kunz R, Vist G, Brozek J (2011). GRADE guidelines: 1. Introduction-GRADE evidence profiles and summary of findings tables. J ClinEpidemiol.

[12] Guyatt GH, Oxman AD, Kunz R, Falck-Ytter Y, Vist GE, Liberati A (2008). Going from evidence to recommendations. BMJ.

[13] Deeks JJ, Higgins JPT, Altman DG (editors). Chapter 9: Analysing data and undertaking meta-analyses. In: Higgins JPT, Green S (editors). Cochrane handbook for systematic reviews of interventions version 5.1.0 (updated March 2011). The Cochrane Collaboration, 2011. Available from www.cochrane-handbook.org.

[14] Sterne JAC, Egger M, Moher D (editors). Chapter 10: Addressing reporting biases. In: Higgins JPT, Green S (editors). Cochrane handbook for systematic reviews of intervention. Version 5.1.0 (updated March 2011). The Cochrane Collaboration, 2011. Available from www.cochrane-handbook.org.

